# Evidence of mutual non-verbal synchrony in learners with severe learning disability and autism, and their support workers: a motion energy analysis study

**DOI:** 10.3389/fnint.2024.1353966

**Published:** 2024-07-11

**Authors:** Devyn Glass, Nicola Yuill

**Affiliations:** ^1^Children and Technology Lab, School of Psychology, University of Sussex, Brighton, United Kingdom; ^2^The Children and Technology Lab, Autism Community Research Network Sussex, School of Psychology, University of Sussex, Brighton, United Kingdom

**Keywords:** interpersonal synchrony, social motor synchrony, autism, neurodivergence, motion energy analysis, severe learning disabilities, intensive interaction

## Abstract

**Introduction:**

Some research indicates that neurodivergent people are less likely than “neurotypical” people to adapt their movements to a partner’s movements to facilitate interpersonal motor synchrony. Researchers therefore suggest synchrony deficits underlie the social differences associated with autism and other neurodivergences. Intensive Interaction (II) is a client-led approach, where Learning Support Workers (LSW) follow the lead of learners to create balanced and reciprocal interactions.

**Methods:**

We aimed to examine the balance of synchrony in learners with autism and Severe Learning Disabilities and their LSWs in a special education college where learners had prior experience with II. Using Motion Energy Analysis, we assessed the degree to which each partner acted as a leader, and hence which partner acted as a follower, during moments of close synchrony.

**Results:**

Overall, learners and LSWs showed higher than chance synchrony. There were no differences in the degree to which each partner led the moments of synchrony, or the amount pairs synchronized with zero-lag, where there was no delay between each partners’ movements.

**Discussion:**

The equal balance of leading and following in the learner and LSW pairs demonstrates that both partners consistently adapted their movements to their partner’s movements to facilitate synchrony. The findings tentatively challenge the notion of a synchrony deficit in autism and suggest synchrony can be present in cross-neurotype pairs in comfortable and engaging conditions. We discuss the potential for client-led, movement-based approaches to support smooth interactions across neurotypes.

## 1 Introduction

Autism is a neurodevelopmental condition typically characterized by differences or difficulties in social interaction and communication, restricted or repetitive behaviors or interests, and sensory processing differences (American Psychiatric Association, 2013). A considerable amount of literature indicates autistic people have difficulties with reciprocal social behaviors, such as collaboration (e.g., van Ommeren et al., 2012), non-verbal turn-taking (e.g., Chiang et al., 2008), joint attention (e.g., Bruinsma et al., 2004), and coordination (e.g., Fournier et al., 2010). These behaviors contribute to smooth social interactions, which require a “dynamic and reciprocal interplay” between one’s own behaviors and the behaviors of others (Dumas and Fairhurst, 2019, p. 2). A growing body of literature terms the natural emerging of simultaneous and coordinated social behaviors Interpersonal Synchrony (IPS). Several terms are used in the literature (e.g., attunement, behavioral alignment/matching/coordination, and mirroring). Here, we use IPS to describe temporally-matched behaviors that occur during interaction. We focus on a specific aspect of IPS, Social Motor Synchrony (SMS), which involves synchronizing non-verbal motor movements with a partner (Fitzpatrick et al., 2016). However, IPS can also include emotions, physiological processes, and thoughts, such as goals or shared understanding (Bernieri and Rosenthal, 1991; Dumas and Fairhurst, 2019).

Interactions that require IPS, such as joint attention and joint action, are recognized as pivotal skills in the development of social and language abilities (Charman, 2003; Cerullo et al., 2021). From an enactivist perspective, social cognition is grounded in the embodied way individuals interact with others and their environment (Holton, 2010; De Jaegher, 2013). An underlying difficulty with IPS has therefore been suggested as a potential contributor to the social interaction differences often observed in neurodivergence (Ramseyer and Tschacher, 2011; Fitzpatrick et al., 2016; Gvirts Problovski et al., 2021). In particular, SMS deficits have been said to underlie social interaction and communication difficulties in autism (Mcnaughton and Redcay, 2020; Zampella et al., 2020). If smooth social exchanges are dependent on a dynamic coupling of behaviors, then a difficulty with SMS may inhibit social-cognitive development and social interaction abilities.

Several studies show lower SMS, henceforth referred to as synchrony, in mixed pairs (an autistic and a non-autistic partner) compared with non-autistic pairs in a range of interactions, such as joint improvisation games, conversations, and experimental paradigms (Brezis et al., 2017; Fitzpatrick et al., 2017; Koehler et al., 2022). It has been suggested that the lower synchrony observed in mixed-dyads evidences impaired synchrony in the autistic partner (Mcnaughton and Redcay, 2020). However, synchrony is a product of an interaction, which requires each partner to engage and adapt their behavior to their partners’ behavior and allows co-construction of intersubjectivity (Laursen and Hartup, 2002; Milton, 2012).

López (2015) argues that traditional theories consider autism as an individual condition, independent from the social context, despite being characterized by social interaction differences or difficulties. A growing body of literature has begun to adopt a second-person approach to the study of social development and social neuroscience. Second-person approaches suggest the mechanisms underlying social interaction fundamentally differ from the mechanisms involved in social observation (Redcay and Schilbach, 2019). Moore and Barresi (2017) also propose several forms of information only occur during interaction, including several which are central to IPS, such as contingency, reciprocity, shared intentions, and affective engagement. It is therefore necessary to consider interaction, and thus synchrony, from an interpersonal framework. This means taking into account the contribution of each individual’s traits to the interaction, and the similarities or differences between social partners (Bolis and Schilbach, 2018).

Milton’s (2012) Double Empathy Problem has allowed several complimentary accounts of mutual misunderstanding to come to the foreground. Georgescu et al. (2020) refer to these accounts under the Interactional Heterogeneity Hypothesis (IHH), which emphasizes the difference in autistic and non-autistic people’s perceptions and experiences of the world. In interaction, this divergence can result in misunderstandings and misrepresentations of partners of the other neurotype (for a summary, see Georgescu et al., 2020). The IHH predicts that social interaction difficulties arise due to an interpersonal mismatch, as opposed to social interaction impairments in the autistic partner.

Emerging findings support the IHH. Cross-neurotype interactions are said to be more complicated or different than interactions with someone of the same neurotype (Wilson and Bishop, 2021). Autistic people describe feeling more comfortable interacting with other autistic people, and experience smooth conversational exchanges (Crompton et al., 2020). Additionally, both autistic and non-autistic people report greater affiliation with, and a preference for interacting with, people of the same neuro-type or people with similar characteristics, such as autistic traits (Sasson et al., 2017; Morrison et al., 2019; Bolis et al., 2021). Together, these findings suggest people of the same neurotype can feel more closely aligned than mixed-neurotype pairs.

Synchrony is also associated with rapport and perceived social unity (Tickle-Degnen and Rosenthal, 2009; Au and Lo, 2020), and can be disrupted by discomfort with one’s social surroundings. Asher et al. (2020) found lower social-motor synchrony and heart rate synchrony in interactions involving socially-anxious partners compared with non-socially anxious partners. Some autistic people need time to build rapport with new people (Robledo et al., 2012; Scott-Barrett et al., 2019). They can also take longer to habituate to new environments than non-autistic people and can have sensory processing differences, which could potentially limit the ease of social interactions and hinder synchrony (Vivanti et al., 2018; Jamal et al., 2021). Despite this, most synchrony studies have involved unfamiliar partnerships or have been situated in unfamiliar environments, such as university study centers (e.g., Fitzpatrick et al., 2017; Georgescu et al., 2020; Liu et al., 2021). Glass and Yuill (2023) found autistic pairs displayed similar synchrony to non-autistic pairs in carefully considered social contexts: in familiar settings and partnerships with personalized tasks. It remains unclear whether the lower synchrony previously observed in mixed pairs (e.g., Georgescu et al., 2020) is the result of an interpersonal mismatch, or whether this is influenced, and may be improved, by other contextual factors.

The impact of frequent interactional misunderstanding is arguably greater for autistic or neurodivergent people than for neurotypical people (Mitchell et al., 2021). Misunderstanding creates a barrier for autistic people to participate in embodied interaction, which provides the foundation for social cognition (Fuchs and de Jaegher, 2009). Crucially, since social interaction allows us to develop an “implicit understanding of others” (López, 2022, p. 369), reduced opportunities for positive interactions across neurotypes can prevent us learning how to interact with people who have different interaction styles and can perpetuate a negative view of people who interact differently to ourselves (Mitchell et al., 2021). Siegel-Causey and Bashinski (1997) proposed engineering social contexts to improve communication between partners when one partner communicates differently. They emphasized the importance of approaches that enhance communication of both interaction partners.

One way to enhance communication in cross-neurotype interactions is through IPS. Learner-led synchrony, where a parent or caregiver follows the lead of an infant to create synchronous, playful exchanges is a key feature of parent-infant interaction (Leclère et al., 2014). It is crucial for development of communication and social adaptation, self-regulation, and empathy (Feldman, 2007 2014). The process of engaging in shared embodied interactions also supports intersubjectivity and helps build trust and rapport (Laroche et al., 2014). These early interaction patterns provide a basis from which to understand the development of connected social interactions and offer a process for improving communication and synchrony in mixed-neurotype interactions.

Intensive Interaction (II) is an interpersonal approach for working with neurodivergent learners, particularly those with Severe Learning Disability (SLD) whose communication is often not understood. Synchrony and contingent movement are central to II. Based on the parent-infant interaction literature, the II practitioner’s role is to follow the learner and communicate in “their language” to build connected exchanges and nurture relationships (Hewett et al., 2011). Reciprocal interactions are created via short, playful, and synchronous interactions, for example, by echoing the learner’s vocalizations and movements to offer a complementary, communicative response (Delafield-Butt et al., 2020). Improvements have been seen in reciprocal non-verbal interaction between neurodivergent learners and II practitioners within minutes of starting II (Zeedyk et al., 2009; Delafield-Butt et al., 2020). Similar results are found in other approaches that employ learner-led synchrony, including Dance and Movement Therapy (DMT), and Improvisational Music Therapy (IMT). For example, improvements in synchrony have been observed over time between music and dance therapists and autistic children (Koehne et al., 2016; Dvir et al., 2020), which can generalize to other contexts and relationships (Venuti et al., 2017).

Using observational coding methods, improvements in reciprocity following II have been attributed to an increase in learners’ social engagement and responses to practitioners’ behaviors (Zeedyk et al., 2009). However, studies using similar methods indicate autistic partners may be less likely to adapt their movements to facilitate synchrony than non-autistic partners, even following learner-led, movement-based interventions. Using the Kestenberg Movement Profile (KMP), Dvir et al. (2020) found that an increase in synchrony between a music therapist and an autistic child was driven by the therapist better adapting their movements to the child’s movements over time, but the autistic child’s degree of adaptation did not change. The KMP is a well-established observational coding system to identify patterns of synchrony, which is used extensively to analyse movement patterns in music and movement-based therapies. However, it relies on manual coding from observations of movement rhythms. Coders who have received 45 h of training, just 15 h fewer than Dvir et al.’s (2020) coders, were found to have inconsistent reliability (Koch et al., 2001). More objective, automated methods are available to measure synchrony (Paxton and Dale, 2013). Frame-Differencing-Methods (FDMs), for instance, allow full-body motion capture, which Paxton and Dale (2013) argue are one of the most effective techniques to measure synchrony as they recognize the dynamic and fluid nature of IPS. FDMs offer an inexpensive way of capturing full-body motion that is comparable to more costly 3D motion detectors in terms of robustness and reliability (Dunbar et al., 2022). To date, they have not been applied in naturalistic settings to examine which partner leads and which follows between partners of different neurotypes.

This is the first study to examine the balance of non-verbal motor synchrony between mixed-neurotype pairs using automated methods (Motion Energy Analysis) during personalized activities and in familiar contexts. We partnered with a special education college where II was used as a standard approach and investigated the balance of synchrony between neurodivergent learners and their Learning Support Workers (LSWs) by examining the extent to which moments of synchrony were led by the learner, led by the LSW, or that occurred because of simultaneous onset of both partners’ movements.

## 2 Method

This study was approved by the University’s Sciences and Technology Cross-Schools Research Ethics Committee. The data were collected in an independent special education college. Written informed consent for the learners to take part and to be video-recorded was obtained from parents/carers at the start of the academic year in September. Before taking part in the activity, the young people read an appropriate Social Story^™^ with their Learning Support Worker (LSW) and were supported to consider their consent by ticking a box to take part and to be video-recorded. One young person with parent/carer consent took part in the activities but chose not to be video-recorded: they could therefore not be included in the analyses. Participants consented for their images to be used in presentations and publications.

### 2.1 Participants

Ten learners (1 female, 9 male) aged 19–22 years (*M* = 20.18, SD = 0.98) participated in the study and agreed to be video-recorded (Table 1). Parents/carers provided the diagnoses of the young people, which were confirmed by the college with information from learners’ Education Health and Care Plans (EHCPs).^1^ All had diagnoses of Severe Learning Disability (SLD). Eight had diagnoses of autism, one was diagnosed with Williams Syndrome (WS), and one had Worster-Drought Syndrome (WDS), a form of cerebral palsy. Five parents/carers listed additional or secondary diagnoses (Table 1). The college facility provides education, care, and therapy for autistic young people with a high level of support needs who may have additional diagnoses of learning disabilities.

**Table 1:** Participants’ demographic details.

Pair	Learner age	Learner gender	Learner diagnoses	Level completed	LSW gender
1	21	M	Autism, Fragile X Syndrome	L1	Female
2	19	M	Williams Syndrome, Communication Disorder, Severe Learning Disability	L2	Female
3	20	M	Autism, Severe Learning Disability	L1	Female
4	21	M	Autism, Severe Learning Disability	L2	Female
5	20	M	Autism, Severe Learning Disability	L2	Female
6	22	M	Autism, Severe Learning Disability	L2	Female
7	20	M	Worcester-Drought Syndrom, Severe Learning Disability	L2	Male
8	21	F	Autism, epilepsy	L2	Male
9	19	M	Autism, Attention Deficit Hyperactivity Disorder, Sensory Processing Disorder, SLD	L2	Male
10	19	M	Autism, ‘Severe Learning Disability	L2	Female

People with WS, WDS, and autism show similarities in social cognition and communication (Clark et al., 2010; Asada and Itakura, 2012), which Asada and Itakura (2012) argue means approaches to support social interaction and communication may be shared. All learners were therefore neurodivergent. Results for the subset of the sample with a primary diagnosis of autism are presented in the supplementary materials. There were no differences in the results in the eight autistic participants compared with the full sample. We therefore present the results for the full sample.

Each learner took part with a different Learning Support Worker (LSW). Ten LSWs therefore participated (7 female, 3 male). Eight were mixed gender pairs and two were matched-gender pairs. All LSWs received a standard package of training typical for specialized education units in the UK, which included courses from the National Autistic Society. All LSWs had experience of using Intensive Interaction in the college setting.

### 2.2 Materials and procedures

Participants took part in the study during the college day. Each session was video-recorded, after video-recording of the consent procedure.

#### 2.2.1 Chat lab connect

**Figure 1:**
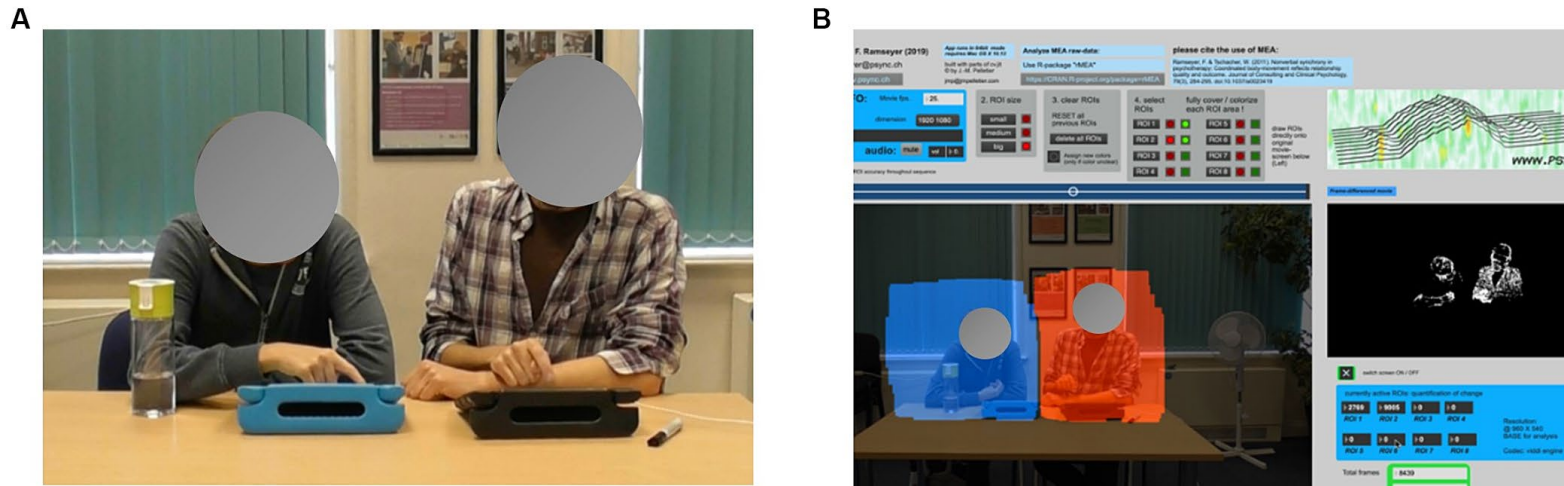
**(A)** One pair sitting side-by-side to play Connect, and **(B)** The Regions of Interest (ROIs) depicted in the MEA software.

**Figure 2:**
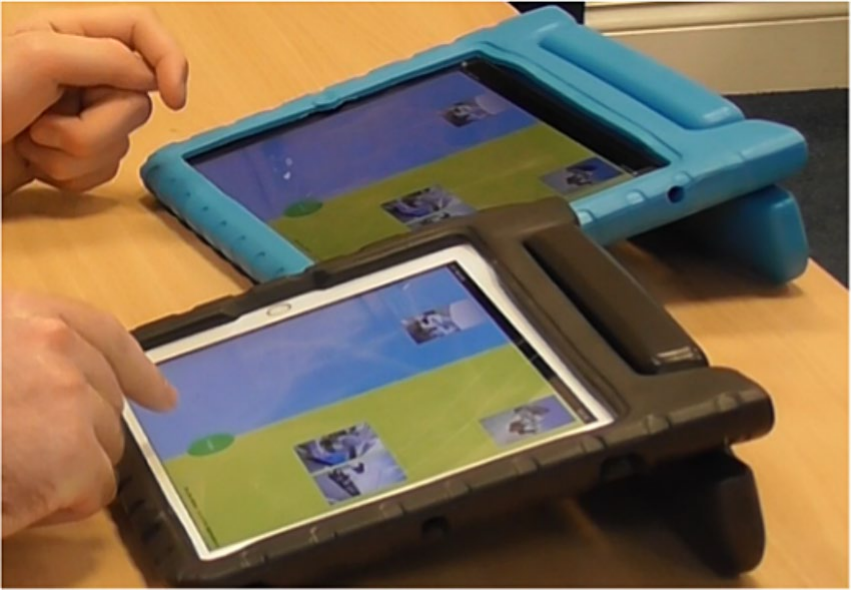
Chat Lab Connect played on two connecting iPads, with Formula 1^™^ cars in one category and rally cars in the second category.

Autistic learners can feel connected with others when engaged in interactions related to their interests (Heasman and Gillespie, 2019; Davey, 2020). Learners and LSWs were therefore asked to play Chat Lab Connect, a picture-sorting activity played on a web app that was personalized to each learners’ interests. The pair sat side-by-side at a table and played Chat Lab Connect (see Figure 1A) developed in the [Children and Technology Lab] at the [University of Sussex] (Holt and Yuill, 2017; Yuill, 2021). Connect is played across two adjacent tablet devices connected via Wi-Fi. Players are required to sort personalized pictures into cells in a grid on their own tablet (see Figure 2). Picture placement must match across both partners’ tablets before the next picture for sorting is made available. There are two levels of difficulty; level 1, “matching” (L1), requires participants to place their pictures in the location on their own tablet corresponding to their partner’s placement. Level 2, “matching and sorting” (L2), requires the pictures to be matched but also correctly grouped according to two objectively pre-defined group categories. For instance, Disney^®^ human characters would be grouped in one column of the grid, and Disney^®^ animal characters would be grouped on another column of the grid. Table 1. summarizes the levels the learners played during their study sessions.

#### 2.2.2 Motion energy analysis

We used Motion Energy Analysis (MEA), an automated program to extract time series data from the video recordings, and the corresponding rMEA package for R Studio to examine synchrony between the learners and LSWs at each time-point (Ramseyer, 2020; Kleinbub and Ramseyer, 2021). MEA uses a Frame-Differencing Method to monitor pixel changes in each video frame. Each partner’s time series are distinguished by pre-defining two Regions of Interest (ROI), which capture the movement of each partner’s upper body (see Figure 1B). We restricted our ROIs to the upper body as their lower body could not be seen when sitting at a table to play the activity.

We then followed Kleinbub and Ramseyer’s (2021) procedure for calculating synchrony in each dyad using rMEA. This first involved calculating windowed cross-lagged correlations (WCLC) of the time series for the two partners in each dyad (Kleinbub and Ramseyer, 2021). We used a maximum lag of ±2 s and selected windows of 10 s with increments of 2 s. There is no current consensus regarding the best parameters to use when calculating WCLC. Instead, it is determined by the researcher’s judgment considering the individual data set and research questions. We selected these parameters to (a) capture information about which partner is leading and which is following, along with matching movements, and (b) use local time-series assessment methods, which analyse the entire time-series by windows. Unlike global methods, local methods are not based on the assumption that one partner leads or influences the other partner for the entire interaction. Instead, it allows for changing interdependence of synchrony across the whole time-series (Schoenherr et al., 2019).

The cross-correlations were standardized to account for different sized ROIs and their absolute values were used to give one overall synchrony score, meaning that positive and negative cross-correlations were incorporated into the overall measure of synchrony. By including positive and negative cross-correlations, both in-phase and anti-phase synchrony are captured by the dyad’s overall synchrony score. This means that identical movements that are performed simultaneously (in-phase) are included as well as movements that are different, but rhythmically matched (anti-phase), such as when one partner leans forward and the other leans backward. This reflects the dynamic nature of spontaneous synchrony (Scheidt et al., 2021).

### 2.3 Analyses

#### 2.3.1 Synchrony in learners and LSWs compared with chance

One possible limitation of WCLC is that synchronous movement observed between the dyads is achieved coincidentally rather than from true interpersonal coordination. To rule out this possibility, we followed Kleinbub and Ramseyer’s (2021) shuffling procedure to calculate a measure of pseudo-synchrony. This involved creating a set of pseudo-dyads who did not interact by pairing a single time-series from a partner in one dyad with a single time-series from a partner in a different dyad. We then calculated overall synchrony scores for this new, random set of dyads using the procedure we described previously and used a *t*-test to compare these to the set of real dyads. If the cross-correlations in the real dyads are more pronounced than in the pseudo-dyads, we can infer the cross-correlations between learners and LSWs were due to genuine synchrony between the two partners (Kleinbub and Ramseyer, 2021).

We then compared the average amount of motion energy in the learners compared with the LSWs. Motion energy scores indicate the amount each individual moved during the study. Similarity of movement quantity in learners and LSWs would support the capacity for the pseudo-dyad analysis to provide true measures of chance synchrony. The session lengths varied from 1 min 48 s to 7 min 39 s. The videos were trimmed to include just the gameplay, starting as the pair first engaged with the device and finishing after the LSW declared “you made it!,” which appeared on the device at the end of the game. We therefore calculated a rate-per-minute (RPM) score for motion energy to account for differences in session lengths. The data violated the assumption of homogeneity of variances, so we used a Wilcoxon Rank Sum test to compare motion energy in learners compared with LSWs.

#### 2.3.2 Synchrony according to pairs’ gender composition and task difficulty

We also examined whether mixed-gender pairs and matched-gender pairs differed in synchrony. We created variables for “gender-match” and “gender-mixed.” The gender-mixed group was not normally distributed, so we used a Wilcoxon Rank Sum test to examine differences in synchrony between pairs whose genders were matched compared with pairs whose genders were mixed. Then, we examined whether synchrony differed according to the level of game difficulty by examining differences in those who completed L1 of Connect (matching) with those who completed L2 (matching and sorted). Four participants played L1 and six played L2. We used a *t*-test to compare differences in synchrony for those who played L1 and L2 of the Connect app.

#### 2.3.3 Balance of leader and follower roles

The MEA program extracts information about the lead–lag relationship. The program calculates a “lead” value for each window of the video-recording and for each partner (Kleinbub and Ramseyer, 2021). This means it is possible to quantify the extent to which each partner leads the synchronous movements. We used these values to create a mean leading score for the learners and LSWs. Following the same process, we extracted the zero-lag data to quantify the extent to which pairs moved synchronously without a lag or delay. This resulted in variables for three synchrony types: learner-led synchrony, LSW-led synchrony, and zero-lag synchrony. To examine the balance of synchrony, we used a within-subjects ANOVA to examine differences for each synchrony type.

#### 2.3.4 Qualitative case studies

To provide contextual information alongside the quantitative synchrony scores, we supplement the findings with observational case studies to describe what is happening in the interaction during moments of close synchrony.

## 3 Results

### 3.1 Synchrony in learners and LSWs compared with chance

**Figure 3:**
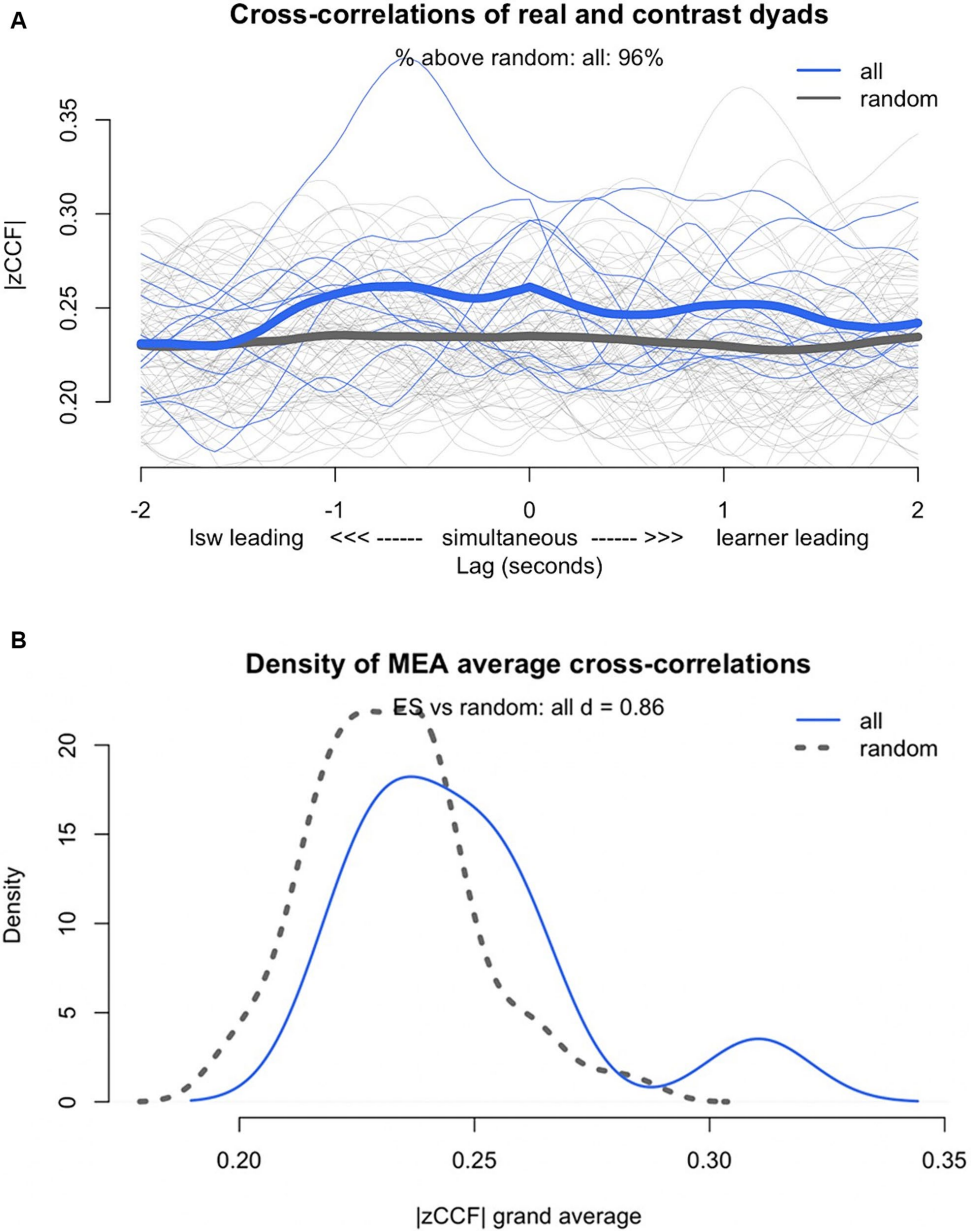
**(A)** Lag plot of Z transformed cross-correlation function (zCCF) for the real (all) compared with pseudo-dyads (random), **(B)** density plot of the Z transformed cross-correlation function (zCCF) for the real (all) compared with the pseudo-dyads (random).

To ensure the synchrony in the participant dyads was not coincidental, we compared the synchrony scores of the real dyads to the synchrony of the pseudo-dyads using the shuffling procedure. The results revealed synchrony was present in the real dyads at a level above chance. The real dyads showed stronger synchrony than the pseudo-dyads did (*t*(9.95) = 1.89, *p* = 0.09, *d* = 0.86, 95% CI[1.53, 0.19]). While there was not a significant difference between the real and pseudo-dyads, there was a large effect size (*d* = 0.86) and 96% of the real dyads’ cross-correlations were stronger than the cross-correlations of the pseudo-dyads (see Figure 3).

To support the real versus pseudo-dyads analyses, we examined the average motion energy in the learners compared with the LSWs. The results revealed no significant difference (*U* = 60, *p* = 0.48, 95% CI[164.56, −57.35]) between the motion energy scores of the learners (Mdn = 146.71, IQR = 189.48) and LSWs (Mdn = 87.71 IQR = 98.42). We can therefore assume the cross-correlations found in the current participants’ interactions are driven by genuine, moment-to-moment interpersonal coordination.

### 3.2 Synchrony according to pairs’ gender composition and task difficulty

We then examined differences in synchrony between pairs whose gender were matched compared with pairs whose genders were mixed. The results revealed no significant differences in synchrony (*U* = 28, *p* = 0.74, 95% CI[0.02, −0.03]) for pairs whose genders were matched (*M* = 0.24, *SD* = 0.03) compared with pairs whose genders were mixed (*M* = 0.24, SD = 0.02). Then, we examined whether synchrony differed according to the level of game difficulty. There were no significant differences in synchrony (*t*(5.91) = −0.47, *p* = 0.66, 95% CI[0.03, −0.04]) between pairs who played L1 (*M* = 0.24, SD = 0.01) or L2 (*M =* 0.25, SD = 0.03).

### 3.3 Balance of leader-follower role

**Figure 4:**
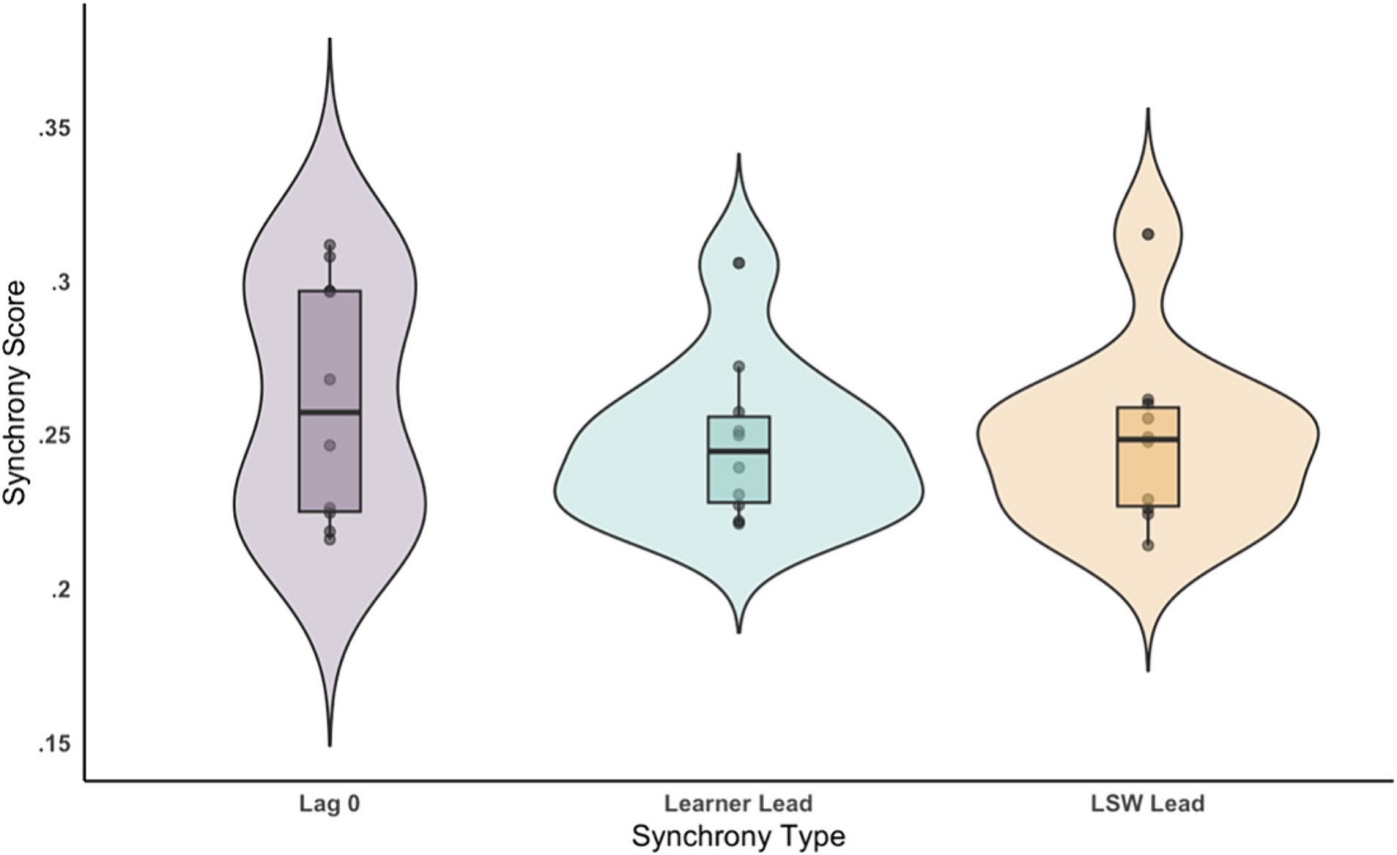
Average synchrony score displayed by pairs for each synchrony type: zero-lag, learner-led, and LSW-led.

Next, we tested for differences between the three types of synchrony: learner-led, LSW-led, and zero-lag synchrony. The ANOVA was not significant (*F*(1, 18) = 1.19, *p* = 0.33, partial *η*^2^ = 0.12), with no differences between learner-led (*M* = 0.25, SD = 0.03), LSW-led (*M* = 0.25, SD = 0.03), or zero-lag (*M* = 0.26, SD = 0.04) synchrony (see Figure 4).

### 3.4 Case study 1: learner following the LSW’s lead

**Figure 5:**
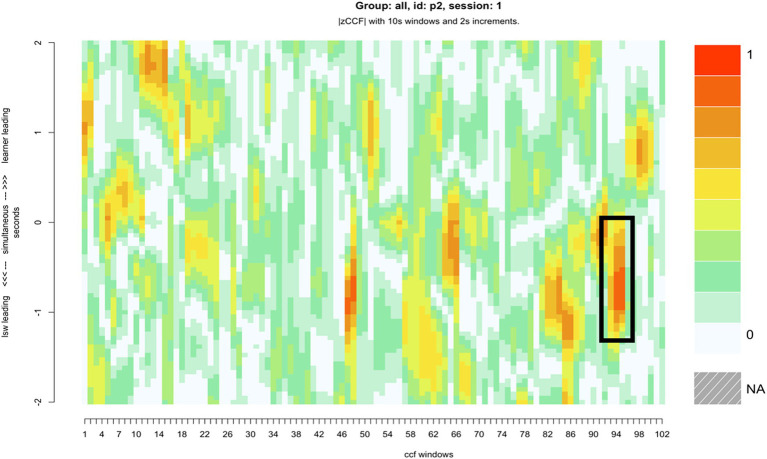
The heatmap for pair 2, with a roughly six-minute period of close synchrony represented, where the LSW led the interaction, and the learner adapted their movements to facilitate synchrony.

**Figure 6:**
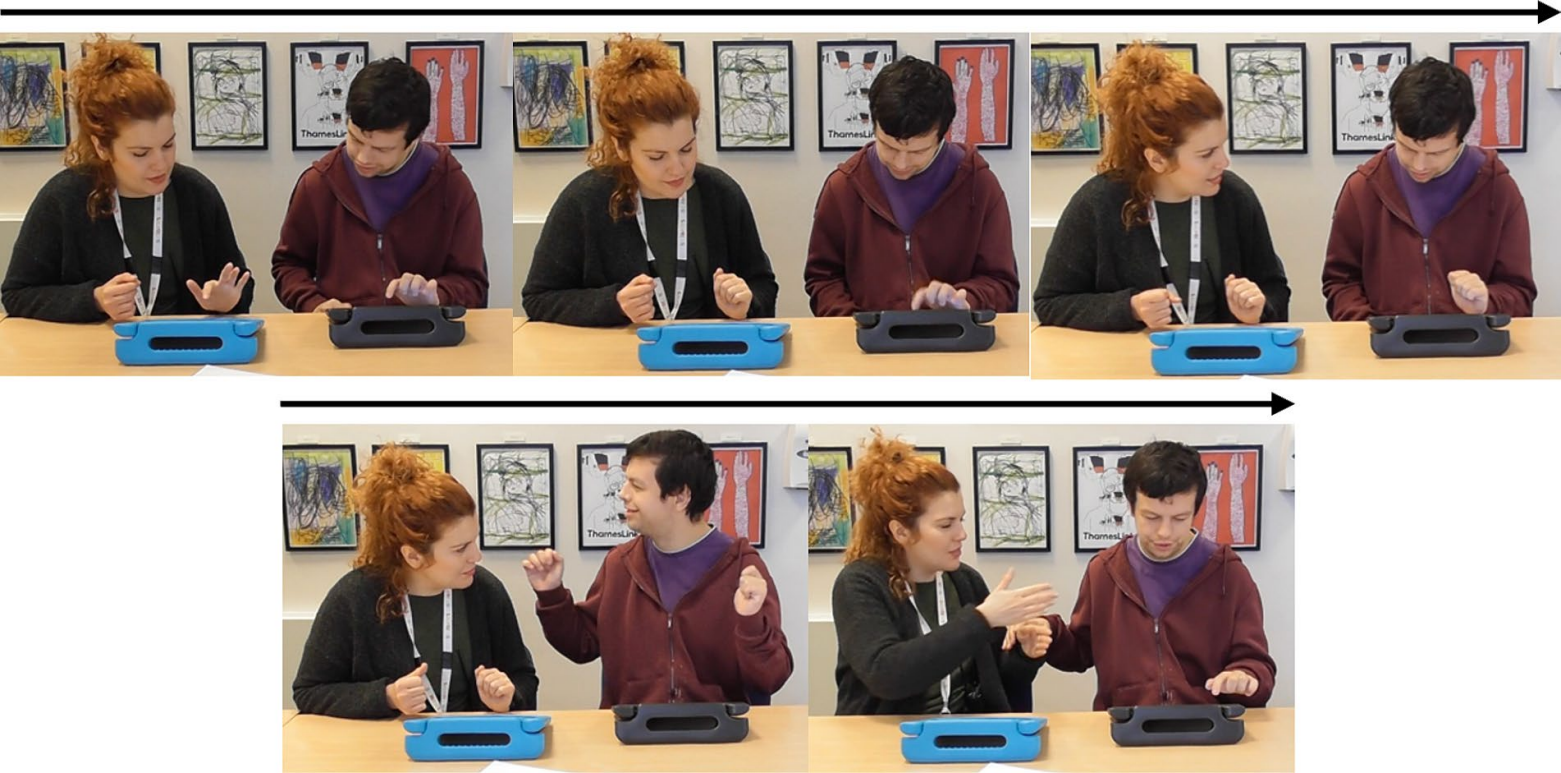
An example of learner 2 (L2) (right) following the LSW’s (left) lead.

Compared with the rest of the sample, pair 2’s overall synchrony scores were average. However, they had among the highest levels of zero-lag synchrony, and the greatest difference between learner-led and LSW-led synchrony (Table 2). For this pair, the moments of close synchrony occurred most frequently when the learner followed the lead of the LSW (indicated by the greater number of darker patches on the lower half of the heatmap compared with the top half) (Figure 5). Figure 5 identifies one point during the interaction when the learner closely followed the lead of the LSW to support synchrony. This is illustrated with stills from the video in Figure 6. At this point, the pair had just placed their final pictures of the game. The LSW pressed “We Agree” and the learner (L2) watched before acting contingently by pressing their own “We Agree” button. The LSW then turned to L2 and exclaimed “We made it, well done!.” L2 joined in with her body movement by turning to face her. The pair then moved synchronously while making celebratory gestures, the LSW says and signs, “Good work!” (Figure 6). L2 appeared to enjoy the Connect game and the interaction with the LSW and frequently attended to the LSW and the LSW’s representation of the game on their iPad. They often responded to the LSW’s verbal initiations, such as “Where is mine?” or “A different windmill!” by turning to face the LSW or their iPad.

**Table 2:** Synchrony scores for each pair, including mean synchrony scores and extent to which the synchrony was led by the learner, the LSW, or that occurred with zero-lag.

Pair	Mean	Learner-led	LSW-led	Zero-lag
1	0.24	0.25	0.22	0.22
2	0.24	0.23	0.26	0.31
3	0.26	0.25	0.26	0.30
4	0.26	0.26	0.26	0.22
5	0.26	0.27	0.25	0.27
6	0.24	0.23	0.25	0.25
7	0.22	0.22	0.23	0.23
8	0.23	0.22	0.23	0.22
9	0.23	0.23	0.21	0.30
10	0.31	0.31	0.31	0.31

### 3.5 Case study 2: consistent following from the LSW and an increase in learner following

**Figure 7:**
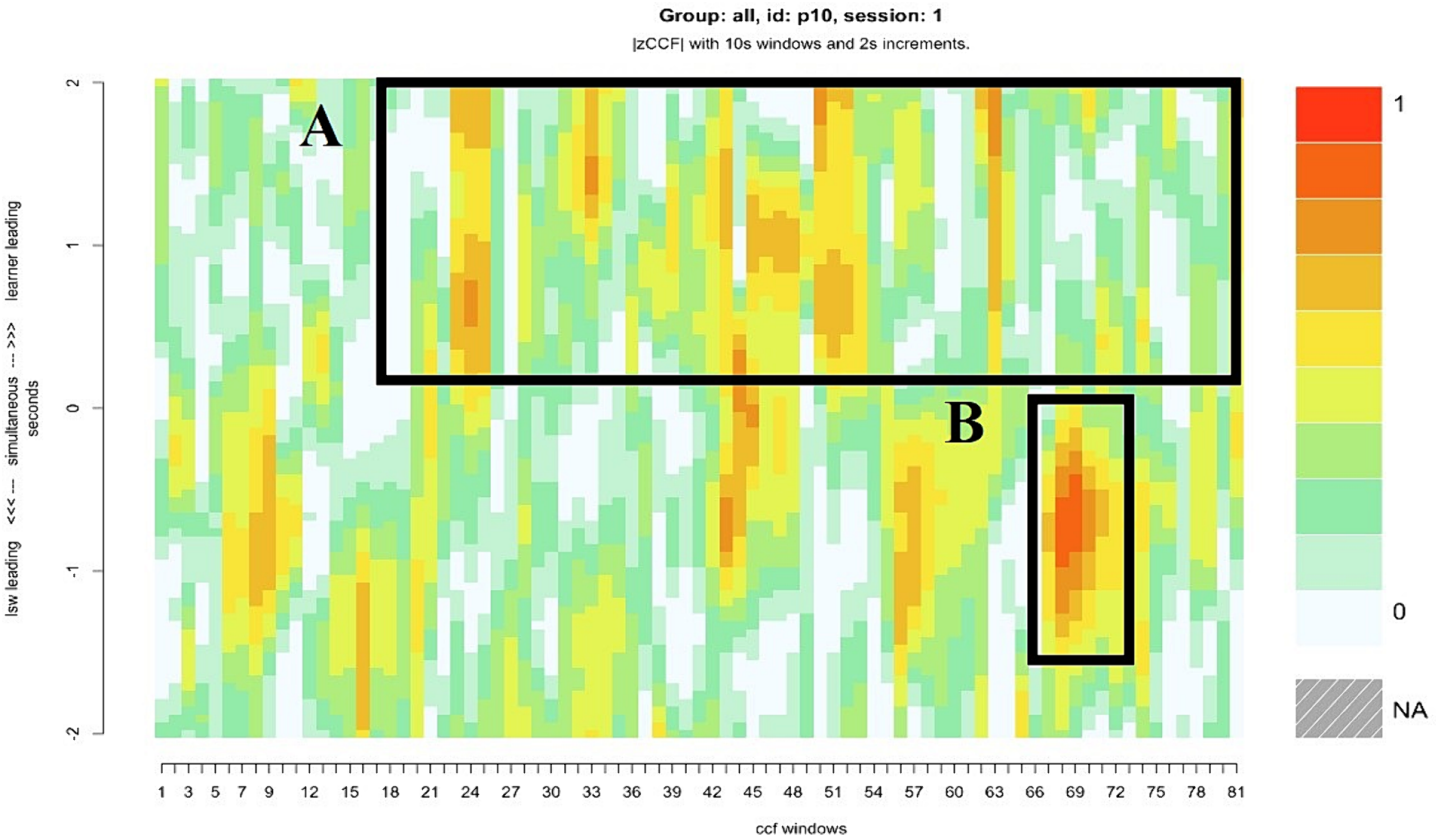
The heatmap for pair 10, who displayed the closest synchrony scores overall. **(A)** The darker patches in the top half of the heatmap indicate periods in the interaction when the LSW followed the lead of the learner, **(B)** the darker patch in the bottom half indicates an instance when the LSW led the interaction.

**Figure 8:**
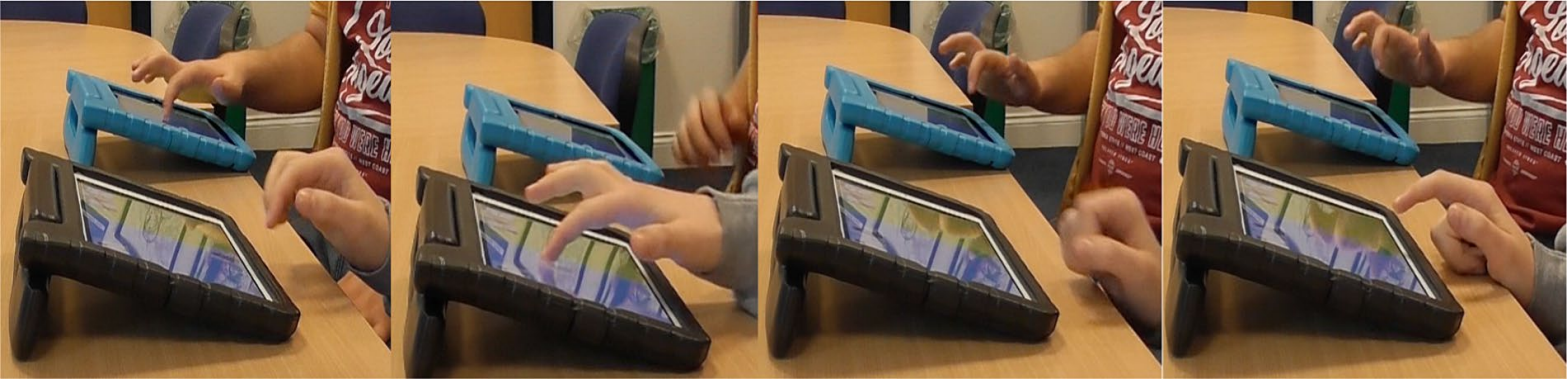
An example of an LSW (right) following the lead of learner 10 (L10) (left) while playing Connect. L10 places their picture while the LSW watches. The LSW places their own picture in the matching location and waits. L10 presses “We Agree,” and the LSW presses their own “We Agree” button immediately afterwards.

**Figure 9:**
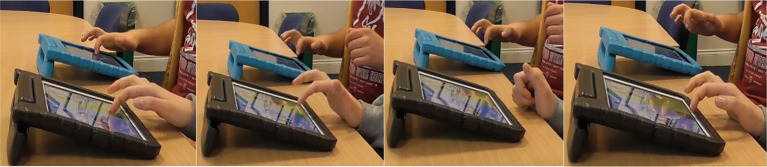
A series of stills illustrating the learner (L10) (left) following the lead of the LSW (right) at the end of the Connect game. L10 follows the LSW’s picture movement. The LSW presses “We Agree,” which is closely followed by L10. When the next picture becomes available, the LSW begins to move the picture first, which L10 then follows.

Pair 10 displayed the most balanced levels of synchrony in the sample. The extent to which the moments of close synchrony were led by the learner and the LSW, or that occurred without a lag, were equal (Table 2). For most of the interaction, the learner’s attention was directed toward their iPad. The LSW was consistently attentive to the learner. For example, their body was turned toward the learner with a friendly, open posture. Figure 7A illustrates the LSW’s consistency following the learner’s lead in the gameplay, the brown and orange patches in the top half of the heatmap indicate several instances where the LSW followed the learner. Typically, they waited for the learner to interact with the game before acting contingently, e.g., by matching the picture placement, or pressing “We Agree” after the learner pressed “We Agree” (Figure 8). Toward the end of the interaction, the LSW led a period of close synchrony (Figure 7B). This means the typical pattern of the LSW following the learner’s lead reversed at the end of the game. Figure 9 illustrates the learner following the LSW’s lead within the context of the Connect game, where the learner moves their picture to match the location of the LSW’s picture.

### 3.6 Case study 3: high zero-lag synchrony

**Figure 10:**
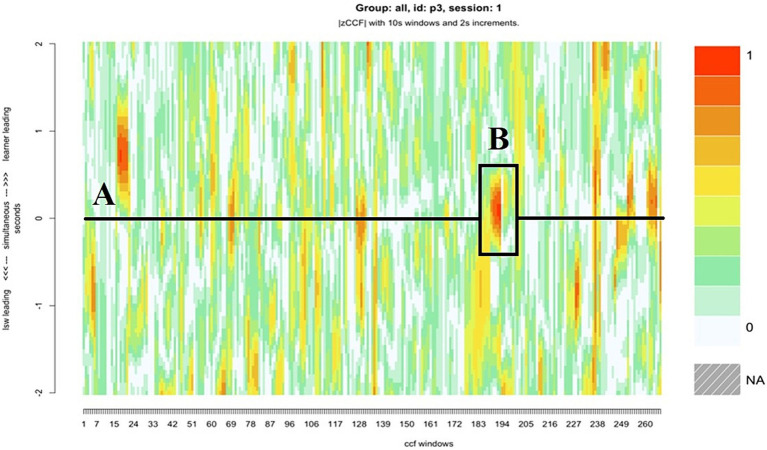
The heatmap for pair 3, who showed several short bursts of close synchrony with zero lag, **(A)** the horizontal line indicates the moments in which synchrony occurred with zero lag, **(B)** the darker patch highlighted indicates one extended moment of close synchrony.

**Figure 11:**
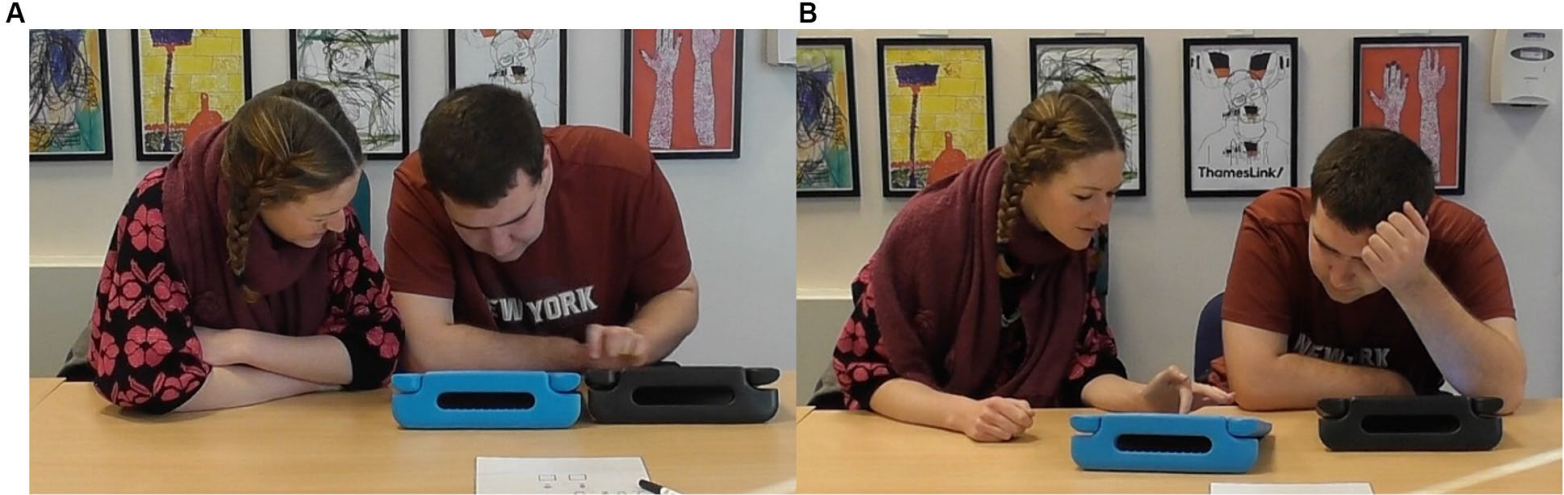
Stills captured from the video of pair 3 where, **(A)** the pairs’ head movements were closely synchronized with little to no lag, and **(B)** the LSW maintained an open and interested posture throughout the interaction.

Pair 3 was one of four pairs who displayed the highest levels of zero-lag synchrony (Table 2). The horizontal black line in the center of the heatmap for pair 3 illustrates several moments where there was little or no delay between each partners’ movements (Figure 10A). These were short, frequent bursts of synchrony, which were characterized by closely coupled, micro-level body movements. For example, during one extended period of close synchrony (Figure 10B), the learner’s and LSW’s head movements were closely synchronized as they looked at the pictures on the left iPad, and then the right iPad (Figure 11A). The LSW in this dyad appeared to match the learner’s posture. Like the LSW in pair 10, they remained turned toward the learner and appeared open and interested in the learner’s actions, with frequent encouraging verbalizations, such as “Oooh!,” “Wow!,” and “Where will you put this one?” (Figure 11B).

## 4 Discussion

This study was the first to use Motion Energy Analysis (MEA) to examine the balance of synchrony in mixed-neurotype pairs in a familiar setting. We measured synchrony that was led by neurodivergent learners or led by their Learning Support Workers (LSWs), and synchrony that occurred with little to no delay between each partners’ movements (zero-lag synchrony). The results revealed synchrony between learners and LSWs at levels higher than chance. Overall, there were no differences in the degree of learner-led, LSW-led, or zero-lag synchrony. This balance demonstrates that both partners mutually adapted their movements to their partner’s movements to facilitate synchrony.

So far, literature suggests autistic people and different interaction partners display weaker synchrony than two non-autistic partners do, which has led several authors to conclude that autistic people have a synchrony deficit (e.g., Mcnaughton and Redcay, 2020). However, we know that synchrony is a relational property, and each partner’s behaviors contribute to the degree of interactional synchrony. It is possible to measure the extent to which each partner facilitates synchrony by examining which partner leads, and therefore which partner follows. Some findings indicate that autistic partners are less likely than non-autistic partners to adapt to, or follow, their partners’ movements to facilitate synchrony (e.g., Delaherche et al., 2013; Marsh et al., 2013; Brezis et al., 2017; Dvir et al., 2020). The current findings, however, suggest some conditions under which autistic people can adapt their movements and facilitate synchrony to an equal degree with their non-autistic partners.

We know autistic people can have sensory processing challenges, and different patterns of attention and social interaction compared with non-autistic people (Murray et al., 2005; Suarez, 2012). Sensory sensitivities can contribute to feelings of anxiety in autism (South and Rodgers, 2017). Since synchrony can be disrupted due to feelings of anxiety and unease (Asher et al., 2020), sensory challenges may have hindered synchrony in previous studies that have taken place in unfamiliar, potentially overstimulating, social contexts. Tasks requiring additional cognitive processing demands in previous research may have also affected the capacity for participants to fully engage with their social partner, preventing the pair from closely synchronizing. In particular, tasks requiring constrained movements or additional cognitive demands may not sufficiently capture the attention and motivation of autistic participants, who can show more localized attention patterns than non-autistic people (Murray et al., 2005; Murray, 2018). For instance, it is possible that elements of previous tasks have drawn the attention of the autistic partner away from the interaction, including constrained movements, such as rocking chair motion (Marsh et al., 2013), explicit imitation of a partners’ movements (Brezis et al., 2017), or following instructions to build a puzzle (Delaherche et al., 2013). Manders et al. (2021) demonstrated that autistic participants are more likely to engage with the task instructions than with a partner during Dance and Movement Therapy (DMT), which influenced the degree to which they followed the therapists’ movements. If an autistic partner’s attention is drawn away from the interaction and toward procedural elements of a task, it is understandable that they would be less likely to follow their partner’s movements to facilitate synchrony. The Connect app, used in this study, is a novel task designed to provide an opportunity for social interaction by scaffolding awareness of a partner and contingent action (Holt and Yuill, 2017; Yuill, 2021). The findings demonstrate above chance synchrony in mixed-neurotype interactions in carefully supported environments.

The Connect app also afforded the opportunity to personalize the content of the game. Autistic participants highlighted barriers to their engagement in Brezis et al.’s (2017) mirror game, citing difficulties with attention and motivation. Our finding that synchrony was higher than chance in mixed-neurotype pairs is consistent with some previous literature that used participant-led conversations or personalized tasks. Romero et al. (2018), for instance, found higher than chance levels of synchrony between autistic children and therapists in child-led conversations. Other studies have shown that synchrony between pairs of autistic children can equal synchrony between pairs of non-autistic children when the settings and partners are familiar, and when tasks are personalized (Glass and Yuill, 2023). The current results contribute to a growing body of literature challenging the idea that autistic people have impaired synchrony and demonstrate the importance of task selection in synchrony research.

Some research has examined synchrony in partnerships presumed to be close, such as autistic children and their parents, revealing weaker synchrony in autistic children and their parents compared with non-autistic children and their parents (Marsh et al., 2013; Fitzpatrick et al., 2016; Zampella et al., 2020; Liu et al., 2021). However, most have taken place in unfamiliar, experimental environments (Marsh et al., 2013; Fitzpatrick et al., 2016; Liu et al., 2021), and all have used prescribed tasks, such as “planning a vacation” (e.g., Zampella et al., 2020). One study used an activity, book sharing, that could incidentally align to some participants’ interests, however, the book selection was not tailored and was used as part of a battery of tasks in a University study center (Liu et al., 2021). This is the first study to examine synchrony in mixed-neurotype dyads in a naturalistic setting and with tasks tailored to the interests of the neurodivergent learners. We tailored the tasks to the interests of learners due to previous literature that indicates that autistic people can feel connected with interaction partners when engaging with their specialized interests (Heasman and Gillespie, 2019; Davey, 2020). Under these conditions, we have demonstrated synchrony between autistic learners and LSWs that is equally facilitated by each partner mutually adapting their movements to their partner’s movements.

We used sensitive methods to examine the patterns of leading and following during moments of synchrony. Some previous studies examining patterns of leading and following during free-flowing interaction have used less precise measures to capture synchrony. Dvir et al. (2020), for instance, used the Kestenberg Movement Profile (KMP) to identify patterns of synchrony. While the KMP is a well-established coding system, observational coding of movement rhythms can lack the reliability and objectivity of automated methods of detection (Koch et al., 2001). A benefit of using MEA is the potential to identify synchrony at the micro-level. Combining such objective measures with qualitative observations enables us to illustrate close moments of synchrony that are only recognizable with software that detects granular changes in motion, such as in Case study 3. It is challenging to identify patterns of contingency for micro-movements through observation alone. Case study 1, for example, demonstrates patterns of contingency where the learner followed the LSW’s movements. This was identifiable from the video during the game-play when the learner pressed “We Agree” after the LSW. However, the learner continued to follow the LSW’s movements while they celebrated completing the game. The pattern of leading and following during the pairs’ celebration was only identifiable via the heatmap. Research examining patterns of synchrony using manual coding methods may therefore have underestimated the extent to which an autistic partner adapted their movements to facilitate synchrony.

We also allowed for changing interdependence of synchrony over time. This recognizes that partners sometimes lead the interaction and sometimes follow their partner’s lead, and that this fluctuates over the course of an interaction (Schoenherr et al., 2019). We identified previous studies indicating that autistic partners are less likely than non-autistic partners to follow their partners’ movements. Of the studies allowing for free-flowing interaction, Delaherche et al. (2013) used an automated method of detection: MEA. However, they examined time-delayed synchrony (i.e., synchrony which occurs after a lag using only one lag score per pair). They compared the combined lag scores of mixed neuro-type pairs to the combined lag scores of two non-autistic partners (Delaherche et al., 2013). A combined lag score does not allow identification of which partner is leading and which partner is following in the interaction. This means a deficit in a tendency to adapt one’s movements to a partner’s movements was attributed to the autistic partner in the mixed-neurotype interaction, despite the lag score containing synchrony that was led by both the autistic and non-autistic partner. By using methods that consider the contribution of both partners, we have demonstrated that neurodivergent partners can and do flexibly adapt their behaviors to the same degree as a neurotypical partner to facilitate synchrony in some circumstances.

## 5 Implications

Studies of communication between pairs of autistic and non-autistic people suggest social exchanges may feel disconnected or less fluid than between two people of the same neurotype (Sasson et al., 2017; Bolis et al., 2018; Heasman and Gillespie, 2019; Crompton et al., 2020). However, the current results suggest certain mixed-neurotype relationships can yield close synchrony. This is not to say autistic people do not find it easier to interact with other autistic people than with non-autistic people, and vice versa. Several autistic people describe experiencing a better connection with people of the same neurotype (Morrison et al., 2019; Crompton et al., 2020). Nevertheless, our findings indicate that certain mixed-neurotype pairs can experience close synchrony in carefully supported environments. While we need more research to determine the environmental and relational factors that can help or hinder synchrony, the results indicate potential to support connectedness between autistic and non-autistic interaction partners through therapeutic approaches, such as Intensive Interaction (II), which harnesses social timing and contingency.

Daniel et al. (2022) propose a model of Rhythmic Relating to improve communication in cross-neurotype pairs by using tailored, rhythmic interactions in playful therapeutic interactions. Elements of this learner-centered approach are seen in existing therapeutic approaches designed to improve the relationship and communication between adults and learners or parents/carers and their children, including II and Video Interaction Guidance. Used as a standard approach at the college in the current study, II involves the LSW following the learner’s lead to create playful, non-verbal communicative exchanges (Hewett et al., 2011). Previous research indicates that II can lead to reciprocal interaction and support rapport development within minutes (Zeedyk et al., 2009; Scharoun et al., 2014; Delafield-Butt et al., 2020). Improvements in synchrony have also been seen following other learner-led approaches, such as Improvisation Music Therapy (IMT), which have been observed to generalize to other contexts and relationships (Venuti et al., 2017). While opportunities for close synchrony in mixed-neurotype interactions may support positive social relationships, previous literature suggests it could also have broader developmental and clinical benefits. Synchrony in early interaction is implicated in social and cognitive development (Charman, 2003; Holton, 2010; De Jaegher, 2013). It is also an important underlying feature of co-regulation, itself necessary for the development of self-regulation, and an important target for support in autism (Silkenbeumer et al., 2016; Erdmann and Hertel, 2019). The current findings offer a tentative suggestion that approaches where non-autistic partners engineer their interaction toward social timing could support synchrony, and may therefore have broad clinical benefits. Daniel et al.’s (2022) model offers a way for the elements of tailored social timing to be integrated into a range of therapeutic approaches for autistic young people, offering guiding principles of interaction.

## 6 Limitations and future directions

This study is the first to use objective methods to measure the balance of synchrony in mixed-neurotype interactions in naturalistic settings. While Frame-Differencing-Methods (FDMs) such as Motion Energy Analysis (MEA) are currently recognized as one of the best available methods to examine synchrony, more research is needed to determine its reliability, particularly with regards to researchers’ selection of parameters, which are currently determined by the researcher’s judgment of the data. Direct comparisons with other methods, such as 3D motion detectors and behavioral coding techniques may provide useful insights.

As is typical in autism research, the study is limited by the small sample size, which reflects the difficulties conducting a study in a specialized education setting. Due to the issue of sample size and real-world constraints requiring the project to start part way through the autumn term, the results regarding the balance of synchrony need to be interpreted with some caution. Improvements in learner engagement, social initiation, and contingent non-verbal expression have been noted following or during just one II session (Zeedyk et al., 2009; Delafield-Butt et al., 2020). Learners will have had at least a months experience of II from the start of the school year. While we did not measure the frequency II was used prior to the study, a potential consequence of looking at such a sample could be that the balance of synchrony observed is the result of broader experience with II. We might not see this balance of synchrony in other mixed-neurotype samples. Further research examining the balance of synchrony at learners’ first experience of II would provide insight into the effect of this approach on granular measures of synchrony between mixed-neurotype dyads.

To better develop our understanding of synchrony, studies in a range of dyads in differenct social contexts are crucial, including comparisons of synchrony in all potential pairings (i.e., two non-autistic, two autistic, and mixed partnerships), neurodivergent learners who have not had experience with II and their support workers, and in relationships chosen or preferred by autistic participants. The current study is among the first to examine synchrony in participants with Severe Learning Disability (SLD) and autism. While previous synchrony research has typically involved autistic participants without SLD, there is an absence of research in the natural environments looking at which partner leads, and which partner follows in interactions with autistic participants. II is most frequently applied in specialized education settings, which means it is unclear whether this approach might also support synchrony in other dyads, such as including autistic young people without SLD. Studies employing similar methods across these partnerships would help us better understand the synchrony autistic people experience in close, connected relationships when they are most at ease. It would also allow identification of aspects of the interaction that might be the focus of research to improve interaction, and understanding, across neurotypes.

As with any preliminary findings, these results need further study. We used the Connect app activity as a personalized task that offered opportunities for social interaction. The Connect app was designed and shown to support contingency in autistic children with learning disabilities (e.g., following a partner’s picture placement, or pressing the “We Agree” button after their partner) (Holt and Yuill, 2017). There is therefore potential for the activity to facilitate synchrony. We see in case study 1, that a moment of close synchrony began when the learner followed the LSW’s action of pressing the “We Agree” button. Similarly, in case study 2, synchrony occurs during patterns of contingency, with the LSW following the learner’s actions. Further research is needed to determine how specific tasks help or hinder synchrony in a variety of settings and partnerships.

## 7 Conclusion

A dominant claim in the synchrony literature is that autistic people display impaired Social Motor Synchrony (e.g., Mcnaughton and Redcay, 2020). This model is largely based on studies demonstrating that autistic participants and their interaction partners, whether autistic or not, show weaker synchrony than between two non-autistic partners (e.g., Kaur et al., 2018; Georgescu et al., 2020; Liu et al., 2021). Some argue this is driven by a lower tendency for autistic people to adapt their movements to their partner’s movements to facilitate synchrony (e.g., Marsh et al., 2013; Brezis et al., 2017; Dvir et al., 2020). Our findings challenge this model. We found synchrony between mixed-neurotypes pairs at levels higher than chance that was equally facilitated by neurodivergent learners and LSWs. The balance of learner-led, LSW-led, and zero-lag synchrony observed here tentatively challenges the notion of a synchrony deficit in autism and highlights the need for further investigation into synchrony in autistic people and a variety of interaction partners in naturalistic settings.

## Data availability statement

The raw data supporting the conclusions of this article will be made available by the authors, without undue reservation.

## Ethics statement

The studies involving humans were approved by University of Sussex Sciences Cross-Schools Research Ethics Committee. The studies were conducted in accordance with the local legislation and institutional requirements. Written informed consent for participation in this study was provided by the participants' legal guardians/next of kin. Written informed consent was obtained from the individual(s) for the publication of any identifiable images or data included in this article.

## Author contributions

DG: Conceptualization, Formal analysis, Investigation, Methodology, Project administration, Visualization, Writing – original draft, Writing – review & editing. NY: Conceptualization, Supervision, Writing – review & editing.

## Footnotes

^1^Education and Health Care Plans (EHCPs) are legal documents, which detail a child’s special education needs based on assessments by multidisciplinary teams including health and education professionals.
