# Supplementary material for: Evidence of mutual non-verbal synchrony in learners with severe learning disability and autism, and their support workers: a motion energy analysis study
Source: Front Integr Neurosci. 2024 Jul 11;18:1353966. doi: 10.3389/fnint.2024.1353966 (PMC11269261; doi:10.3389/fnint.2024.1353966)
Supplement: Supplementary file 1 [file Data_Sheet_1.PDF]

Synchrony in learners and LSWs compared with chance

- The real dyads showed significantly stronger synchrony than the pseudo-dyads did (t(17.62) = 2.20, p = .04, d = .66, 95% CI[1.13, .19]).
- There was no significant difference (U = 34, p = .88, 95% CI[213.44, -39.20]) between the motion energy scores of the learners (Mdn = 68.48, IQR = 94.39) and LSWs (Mdn = 63.99 IQR = 42.58).

Synchrony according to pairs' gender composition and task difficulty

- The results revealed no significant differences in synchrony (U = 4, p = .39, 95% CI[.03, -.09]) for pairs whose genders were matched (M = .24 , SD = .02) compared with pairs whose genders were mixed (M = .26, SD = .03).
- There were no significant differences in synchrony (U = 11, p = .49, 95% CI[.04, -.07]) between pairs who played level 1 (M = .26, SD = .01) or level 2 (M = .25, SD = .04).

Balance of leader-follower role

- There were no significant differences between learner-led (M = .26, SD = .03), LSW-led (M = .25, SD = .03), or zero-lag (M = .26, SD = .04) synchrony (F(2,14) = .47, p = .63, *partial* η^2 = .06).
